# Transcriptomic Analysis of Metformin’s Effect on Bovine Viral Diarrhea Virus Infection

**DOI:** 10.3390/vetsci11080376

**Published:** 2024-08-15

**Authors:** Zeyu Li, Yuanxiu He, Junzhen Chen, Duoliang Ran, Jianbo Yue, Qiang Fu, Huijun Shi

**Affiliations:** 1 College of Veterinary Medicine, Xinjiang Agricultural University, Urumqi 830052, China; 320210056@xjau.edu.cn (Z.L.); fq198505@gmail.com (Y.H.); shihuijunmm@163.com (J.C.); xjrdl7@163.com (D.R.); 2 Xinjiajng Key Laboratory of New Drug Study and Creation for Herbivorous Animals, Urumqi 830052, China; 3 Division of Natural and Applied Science, Duke Kunshan University, Kunshan 215316, China; jianbo.yue@dukekunshan.edu.cn

**Keywords:** bovine viral diarrhea virus, RNA-seq, transcriptome analysis

## Abstract

**Simple Summary:**

We investigated the ability of metformin (Met) to inhibit bovine viral diarrhea virus (BVDV) replication in vitro. We analyzed the transcriptomic profiles of BVDV-infected Madin Darby bovine kidney (MDBK) cells after pretreatment with Met to identify activated or suppressed metabolic and signaling pathways.

**Abstract:**

(1) Background: Bovine viral diarrhea virus (BVDV) causes calf diarrhea, bovine respiratory syndrome, and cow abortion, resulting in substantial economic losses in the cattle industry. Owing to its persistent infection mechanism, BVDV is a major challenge in the treatment of cattle. (2) Methods: To determine how metformin (Met) inhibits the interaction between BVDV and host cells, we treated BVDV-infected cells with Met. We then performed an RNA sequencing (RNA-seq) analysis of Met-treated cells infected with BVDV to identify differentially expressed genes (DEGs). Consequently, the RNA-seq results were validated through real-time quantitative PCR (qPCR). (3) Results: Our analysis revealed 3169 DEGs in the Met-treated cells (Met group) vs. the negative controls (NC group) and 2510 DEGs in the BVDV-infected cells after pretreatment with Met (MetBVDV group) vs. the BVDV-infected cells (BVDV group). The DEGs were involved in MDBK interactions during BVDV infection, as indicated by Gene Ontology (GO) and Kyoto Encyclopedia of Genes and Genomes (KEGG) pathway analyses. The potential interactions of the DEGs were confirmed via a protein–protein interaction (PPI) network. Met treatment induced autophagy signaling activity and the expression of the autophagy-related genes ATG2A, ATG4B, ATG10, and ATG12 in BVDV-infected Met-pretreated cells. (4) Conclusions: We found that the host transcriptomic profile was affected by BVDV infection and Met pretreatment. These findings offer valuable new insights and provide support for future studies on the inhibition of BVDV replication by Met.

## 1. Introduction

Bovine viral diarrhea virus (BVDV) induces persistent infection (PI) in cattle, resulting in not only diarrhea but also reproductive disorders, respiratory diseases, and immunosuppression [1,2]. BVDV infection is a major threat to the global cattle industry, leading to substantial economic losses. This virus is common in cattle worldwide, with high incidence rates that negatively impact reproduction and overall herd health. As a result, BVDV infection has been classified as a Class B infectious disease by the World Organization for Animal Health (OIE), not only in northern and western China [3,4,5] but also in some European countries, such as the UK, and the USA [4]. Therefore, effective methods are needed to control BVDV infection in cattle.

Both inactivated and attenuated vaccines are currently used to control BVDV infection [6], but the control of epidemic BVDV strains in various regions of China is ineffective [7]. Thus, in addition to prevention via vaccination, therapeutic agents should be considered to help control BVDV infection.

Metformin (Met) is a biguanide-derived hypoglycemic agent used to treat patients with type 2 diabetes [8,9]. Studies have shown that Met can be used to prevent hepatitis C virus (HCV) infection [10]. Met was reported to inhibit mitochondrial respiratory chain complex I NADH-Q oxidoreductase [11], resulting in the oxidative phosphorylation and activation of AMPK [12,13]. Met activates type I IFN antiviral activity through the activation of the AMP-activated protein kinase (AMPK) pathway to inhibit the replication of HCV, as well as porcine reproductive and respiratory syndrome virus and dengue virus [14,15].

In this study, we investigated the ability of Met to inhibit BVDV replication in vitro. We analyzed the transcriptomic profiles of BVDV-infected MDBK cells after pretreatment with Met to determine the effects of Met on gene expression levels and to identify activated or suppressed metabolic and signaling pathways.

## 2. Materials and Methods

### 2.1. Cell and BVDV Information

MDBK cells (Chinese Academy of Sciences Type Culture Collection, Shanghai, China) were maintained in Dulbecco’s modified Eagle medium (DMEM; Biological Industries, BI, Kibbutz Beit Haemek, Israel) supplemented with 10% fetal bovine serum (FBS; BI) and 1% penicillin/streptomycin solution (BI) at 37 °C. All research materials were free of BVDV.

BVDV TC, kindly provided by Dr. Duoliang Ran, is a cytopathic (CP) biotype [16]. The 50% tissue culture infective dose (TCID_50_) was calculated via the Reed–Muench method [17].

### 2.2. Cytotoxicity Analysis

Met was dissolved in ddH_2_O and diluted to 0 mM, 5 mM, 10 mM, 15 mM, 20 mM, or 50 mM. MDBK cells (5 × 10^4^ cells/well) were cultured in 96-well plates and underwent pretreatment with Met at 37 °C. Then, 3-2,5-diphenyl tetrazolium bromide solution (5 mg/mL) was added and incubated for 4 h at 37 °C. Thereafter, the cells were washed with phosphate-buffered saline (PBS, pH = 7.0), and 200 μL of dimethylsulfoxide (DMSO) was added to each well. The optical density (OD) was read with a multiwell microplate reader at 570 nm. MDBK cell viability is expressed as a percentage of that of the negative control (NC) group. This experiment was performed in the dark.

### 2.3. BVDV dsRNA Detection

Coverslips were placed in 24-well plates, followed by the addition of the cells (1 × 10^4^ MDBK cells/well) and pretreatment with 0 mM, 5 mM, 10 mM or 20 mM Met for 8 h at 37 °C. The cells were subsequently infected with 10^3^ TCID_50_ BVDV at 24 h. The cells on the coverslips were permeabilized with 0.1% (*v*/*v*) Triton X-100 for 5 min at 4 °C, incubated with dsRNA antibodies (1:1200) in PBS overnight at 4 °C, and then incubated with the secondary antibody, donkey anti-mouse CoraLite 488 IgG (H + L) (1:250), for 2 h at room temperature. This experiment was performed in the dark.

### 2.4. Quantitative Real-Time PCR (qPCR)

The cells underwent pretreatment with 10 mM Met for 8 h and were infected with 10 µL of BVDV at 10^3^ TCID_50_ for 0 h, 12 h, 24 h, 36 h, or 48 h. Total RNA was transcribed to cDNA with cDNA Synthesis SuperMix (TaKaRa Bio, Otsu, Japan). The qPCR primers used were designed according to GenBank (NC_001461.1): (F: CCTAGCCATGCCCTTAGTAGGACT; R: GGAACTCCATGTGCCATGTACA, 267 bp). qPCR was performed in a final volume of 20 μL. The reaction conditions were 95 °C for 15 s, followed by 40 cycles of 95 °C for 10 s and 60 °C for 30 s, performed on an ABI 7500 Fast system.

### 2.5. Viral Titer

The collected cells were treated with 10 mM Met for 8 h, infected with 10^3^ TCID_50_ BVDV, and observed at 0 h, 12 h, 24 h, 36 h, and 48 h with an inverted microscope. The Reed–Muench method was used to calculate the TCID_50_ for 7 days in 96-well plates (5 × 10^4^ MDBK cells/well).

### 2.6. Virus Replication Cycle Assay

For the viral inactivation assay, 1 × 10^5^ MDBK cells/well underwent pretreatment with Met (10 mM) or DMSO and were infected with BVDV (10^3^ TCID_50_) at a volumetric ratio of 1:1 at 37 °C for 2 h. Culture medium was collected and ultracentrifuged at approximately 1100× *g* for 1.5 h at 4 °C in 20% sucrose buffer. The virus was resuspended in culture medium prior to incubation at 37 °C for 2 h. After culturing, we removed the virus culture medium. The cell lysates were treated with TRIzol^®^ Reagent (Invitrogen, Carlsbad, CA, USA) for qPCR.

For the viral attachment assay, MDBK cells underwent pretreatment with 10 mM Met or DMSO for 1 h and infected with 10^3^ TCID_50_ BVDV at 4 °C for 2 h. After being washed with PBS, the cell lysates were extracted with TRIzol^®^ Reagent.

For the viral internalization assay, MDBK cells were infected with 10^3^ TCID_50_ BVDV at 4 °C for 1 h and then washed with PBS and 2% FBS in DMEM containing 10 mM Met or DMSO as the culture medium at 37 °C for 1 h. The cells were subsequently washed with PBS. The cell lysates were incubated with TRIzol^®^ Reagent.

For the viral replication assay, MDBK cells were infected with 10^3^ TCID_50_ BVDV at 37 °C for 1 h and then washed with PBS three times. Then, 2% FBS containing 10 mM Met or DMSO was added to the MDBK cells, which were subsequently incubated at 37 °C for 2 h. The cell lysates were incubated with TRIzol^®^ Reagent.

For the virus release assay, MDBK cells were infected with 10^3^ TCID_50_ BVDV at 37 °C for 1 h. After three washes, the samples were incubated in 2% FBS in DMEM for 10 h and washed three times with PBS, and 10 mM Met or DMSO was added for incubation at 37 °C for 2 h. The supernatant was collected and examined via qPCR.

### 2.7. Sample Collection

MDBK cells were treated with Met for 12 h and inoculated with 10^3^ TCID_50_ BVDV for 2 h. The four groups were as follows: uninfected NC cells, Met-treated uninfected cells (Met), untreated BVDV-infected cells (BVDV), and Met-treated BVDV-infected cells (MetBVDV). The cells infected with BVDV for 24 h were collected and subsequently lysed with TRIzol. RNA integrity was assessed with the RNA Nano 6000 Assay Kit of the Bioanalyzer 2100 system (Agilent Technologies, Santa Clara, CA, USA). These experiments were performed with three biological replicates.

### 2.8. Preparation of Samples for Transcriptomic Sequencing

The RNA samples were prepared from a total of 1 μg of RNA per sample. Briefly, mRNA was purified from total RNA. Random hexamer primers and M-MuLV reverse transcriptase (RNase H^−^) were used for first-strand cDNA synthesis. DNA polymerase I and RNase H were subsequently used for second-strand cDNA synthesis. DNA fragments were prepared for hybridization after the adenylation of the 3′ ends. cDNA (370–420 bp) was selected via the AMPure XP system (Beckman Coulter, Beverly, MA, USA). Phusion high-fidelity DNA polymerase, universal PCR primers, and the Index (X) primer were used for PCR. The quality of the PCR products was assessed with an Agilent Bioanalyzer 2100 system. After cluster generation, the library was sequenced on the Illumina NovaSeq platform, and 150 bp paired-end reads were generated.

### 2.9. Quality Control and Read Mapping to the Reference Genome

The raw data (raw reads) were processed via an internal Perl script. Clean data (clean reads) were obtained by removing read data containing the adapter, read data containing poly-N sequences, and low-quality read data from the original data. The Q20, Q30, and GC contents were calculated for the clean data, and all the downstream analyses were based on high-quality clean data. An index was built for the reference genome via HISAT2 (v2.0.5).

### 2.10. Quantification of Gene Expression Levels

The reads mapped to genes were counted via Feature Counts v1.5.0-p3. The length and number of reads mapped to the gene were calculated from the expected fragments per kilobase of transcript sequence per million (FPKM) values. The effects of depth and gene length were considered simultaneously via FPKM analysis.

### 2.11. Differential Expression Analysis

Analysis was performed for two conditions/groups (two bioreplicates per condition) with the DESeq2 R package (1.20.0). The data analysis was performed with DESeq2. The resulting *p* values controlling the error discovery rate were adjusted by Benjamini and Hochberg corrections. Genes are represented by adjusted *p* values.

### 2.12. Gene Ontology (GO) and Kyoto Encyclopedia of Genes and Genomes (KEGG) Enrichment Analysis

The DEGs were subjected to GO enrichment analysis with the clusterProfiler R package, and the gene length bias was corrected. DEGs (*p* < 0.05) were assigned to GO terms. Database resources were obtained from the KEGG database. The statistical enrichment of DEGs in KEGG pathways was tested with the clusterProfiler R package.

### 2.13. Gene Set Enrichment Analysis (GSEA)

A predefined gene set was used to determine significant consistent differences between two biological states via GSEA, a computational approach. Subtle expression differences were revealed via GSEA. GO analysis was performed with a local version of the GSEA tool https://www.gsea-msigdb.org/gsea/index.jsp (accessed on 14 August 2023), and the KEGG dataset was used independently for GSEA.

### 2.14. Protein–Protein Interaction (PPI) Analysis

The PPIs of the DEGs were analyzed with the Cytoscape 3.8.2 database.

### 2.15. Validation of the Transcriptome Sequencing Data

Met and MetBVDV cells were used as samples to validate the DEGs via qPCR. cDNA Synthesis SuperMix (TaKaRa Bio, Otsu, Japan) was used for RNA transfer. The qPCR primers for twelve random DEGs were designed by GenScript https://www.genscript.com/tools/real-time-pcr-taqman-primer-design-tool (accessed on 14 August 2023) (Table 1). Twelve random DEGs [solute carrier family 34 member (SLC34A2), fibroblast growth factor 21 (FGF21), al-dehyde dehydrogenase 1 family member L2 (ALDH1L2), tribbles pseudo kinase 3 (TRIB3), plexin A2 (PLXNA2), epithelial splicing regulatory protein 1 (ESRP1), gap junction protein alpha 1 (GJA1), matrix metallopeptidase 13 (MMP13), nicotinate phosphoribosyl transferase (NAPRT), serpin family D member 1 (SERPIND1), NADPH oxidase organizer 1 (NOXO1) and matrix metallopeptidase 9 (MMP9)] were identified and validated via qPCR, with a final volume of 20 μL, containing 10 μL of SYBR qPCR Master Mix (Qiagen Biotech, Hilden, Germany), 0.8 μL each of forward and reverse primers (10 μM), 1 μL of cDNA, and 7.4 μL of ddH_2_O. The reaction conditions used were 95 °C for 15 s and 40 cycles of 95 °C for 10 s and 60 °C for 30 s, which were performed on an ABI 7500 Fast system. GAPDH was used as an endogenous reference gene. qPCR was performed in triplicate, the 2^−ΔΔCt^ method was used to compare the results with those for the GAPDH gene, and the levels of the genes were evaluated.

### 2.16. Transcriptome Profile Submission

The DEGs and raw files from high-throughput sequencing in this study were submitted to the NCBI Sequence Read Archive (SRA) database (PRJNA766827).

### 2.17. Statistical Analysis

All the data were analyzed with GraphPad Prism 7 (GraphPad Software, Inc., Boston, MA, USA). The asterisks indicate statistical significance according to the Student’s *t* test (*, *p* < 0.05; **, *p* < 0.01).

## 3. Results

### 3.1. Met Served as a Novel Inhibitor of BVDV Replication In Vitro

**Figure 1:**
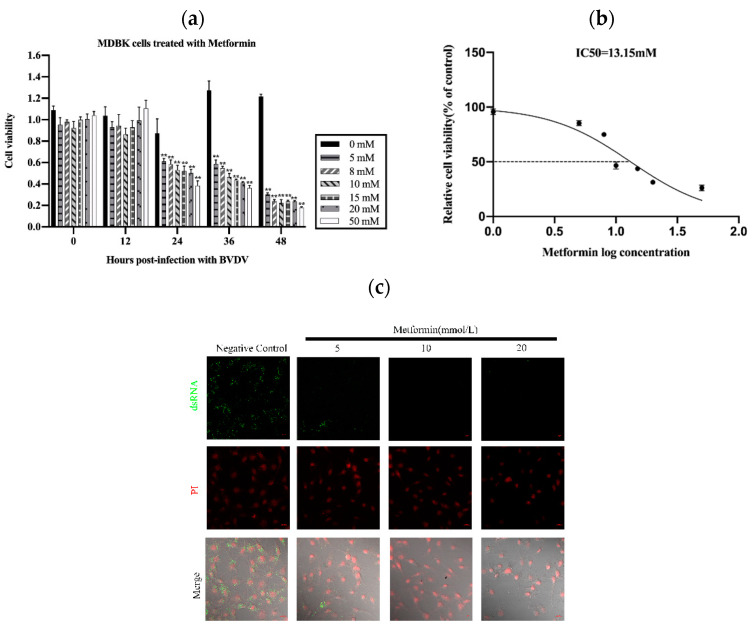

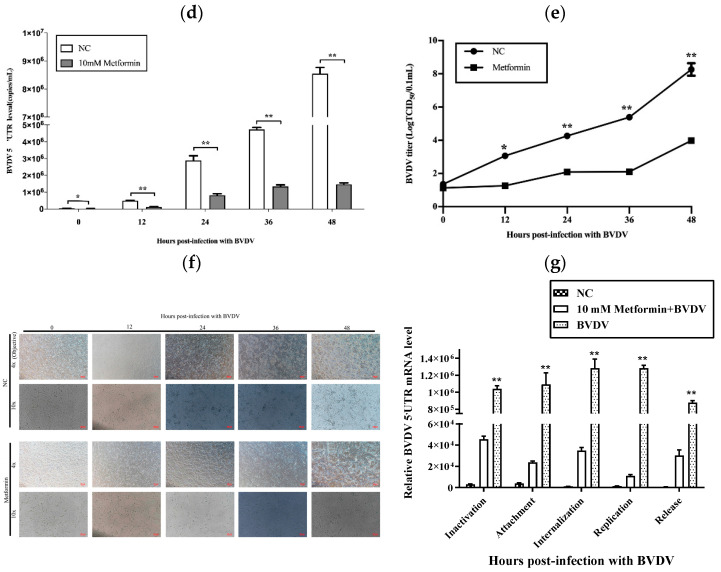
Met influenced the proliferation of MDBK cells. (**a**): Effects of Met at different concentrations (0 mM, 5 mM, 8 mM, 10 mM, 15 mM, 20 mM and 50 mM) on the proliferation of MDBK cells after 48 h. (**b**): The IC_50_ was calculated on the basis of the results in 1a. (**c**): We treated BVDV-infected cells with Met at 0 mM, 5 mM, 10 mM and 20 mM for 8 h. For measurement of the level of BVDV dsRNA by immunofluorescence staining, MDBK cells were infected with 10^3^ TCID_50_ BVDV, with uninfected cells used as a negative control. (**d**): MDBK cells were infected with 10^3^ TCID_50_ BVDV, with uninfected cells used as a negative control. (**e**): Met (10 mM)-pretreated MDBK cells infected with 10^3^ TCID_50_ BVDV were assessed via the Reed–Muench method. (**f**): Met (10 mM)-pretreated MDBK cells infected with 10^3^ TCID_50_ BVDV, with uninfected cells used as a negative control. (**g**): Met (10 mM)-pretreated MDBK cells were infected with 10^3^ TCID_50_ BVDV, and qPCR analysis of the effects of Met on BVDV and its inhibitory effects on the attachment, internalization, replication, and release stages of the BVDV replication cycle was performed. The asterisks indicate statistical significance according to the Student’s *t* test (*, *p* < 0.05; **, *p* < 0.01).

To assess the cytotoxicity of Met in MDBK cells, we treated the cells with various concentrations of Met for various periods. As shown in Figure 1a,b, the viability of the MDBK cells treated with Met for 24 h was reduced (** *p* < 0.01) (Figure 1a). The viability of the MDBK cells was decreased by Met in a concentration-dependent manner (IC_50_ = 13.15 mM) (Figure 1b).

We then used a dsRNA antibody to determine the effect of Met on BVDV replication and assessed the replication of BVDV mRNA in MDBK cells. We also assessed the anti-BVDV activity of Met in MDBK cells. The results showed that Met inhibited viral generation from 12 h to 48 h post-infection (pi) (*p* < 0.01), as demonstrated by viral titration assays (Figure 1c) and qPCR (Figure 1d). At 24 h, the BVDV dsRNA level was reduced by Met (5 mM, 10 mM and 20 mM), and 10 mM Met significantly reduced the number of BVDV dsRNA puncta. These results indicated that Met inhibited BVDV infection in MDBK cells.

We next investigated whether Met inhibits BVDV replication by reducing the cytopathic effect (CPE) caused by BVDV in MDBK cells. We pretreated MDBK cells with Met. At 12 hpi, cell death and the number of syncytia were significantly reduced (Figure 1f).

To explore the effect of Met on the BVDV replication cycle, we performed a qPCR analysis of BVDV mRNA to examine the impact of Met on the attachment, internalization, replication, and release stages of the BVDV replication cycle. Met significantly inhibited the replication stage (Figure 1g). During the internalization and attachment stages, the amount of BVDV mRNA obviously decreased in response to Met, and this effect continued until the release stage.

These data indicated that Met significantly inhibited BVDV mRNA expression and that BVDV infection resulted in the development of a syncytial plaque formation of MDBK cells, which was reduced by Met, particularly at 24 h, 36 h, and 48 h pi. Met inhibited BVDV at the attachment and replication stages.

### 3.2. Evaluation of Transcriptomic Sequencing Data

**Figure 2:**
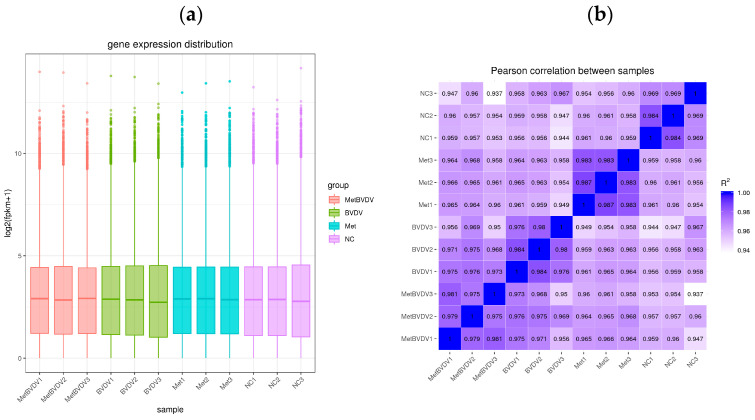
Evaluation of the transcriptome sequencing data. (**a**): The abscissa is the sample name, and the ordinate is the log2 (FPKM+1) value. The box plot of each area represents five statistical values (shown from top to bottom are the maximum value, the upper quartile, the median, the bottom quartile, and the minimum value). (**b**): Heatmap of correlations between samples; the abscissa and ordinate in the figure are the squares of the correlation coefficients of the samples.

To ensure that the sequencing results had biological and statistical significance, we ensured the accuracy and relevance of the results. We performed three biological repetitions of every sample and obtained 4.2 Gb of available data. The Q20 was greater than 97% for the clean data, and the GC content was between 53.05% and 55.77% (Table 2). Gene expression levels from RNA-seq are generally expressed as FPKM values, which are influenced by depth and length; therefore, FPKM values can be normalized to sequencing depth and gene length. After calculating the expression levels (FPKM values) of all genes in each sample, we used box diagrams to visualize the different samples and gene expression levels (Figure 2a). These values are important indicators of the relationship of the sample for which gene expression levels were measured and the accuracy of the measurement.

The encoding scheme suggested a Pearson correlation coefficient (R^2^) greater than 0.92 (under ideal sampling and experimental conditions). On the basis of the FPKM values between samples within and between tissues, a heatmap was drawn, which visually shows the differences between samples within groups and the reproducibility of sample groups. The correlation coefficient between samples indicates that their expression patterns are more similar. All the samples in this study presented correlation coefficients greater than 0.94 in the heatmap (Figure 2b). These results indicated that the samples presented similar gene expression levels and accuracies.

### 3.3. Differential Gene Expression Analysis

**Figure 3:**
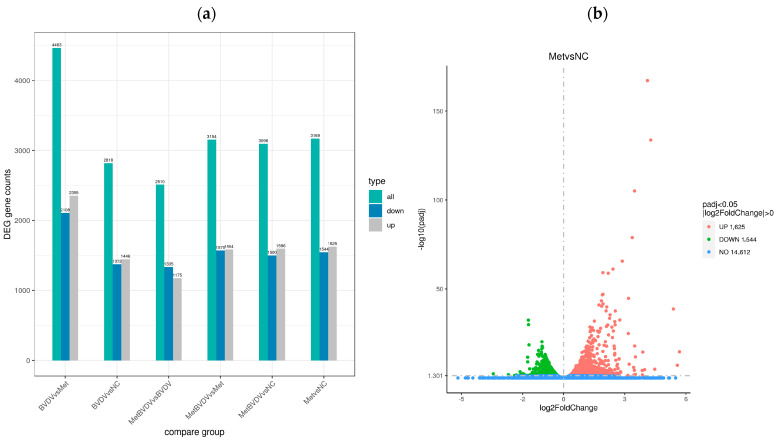

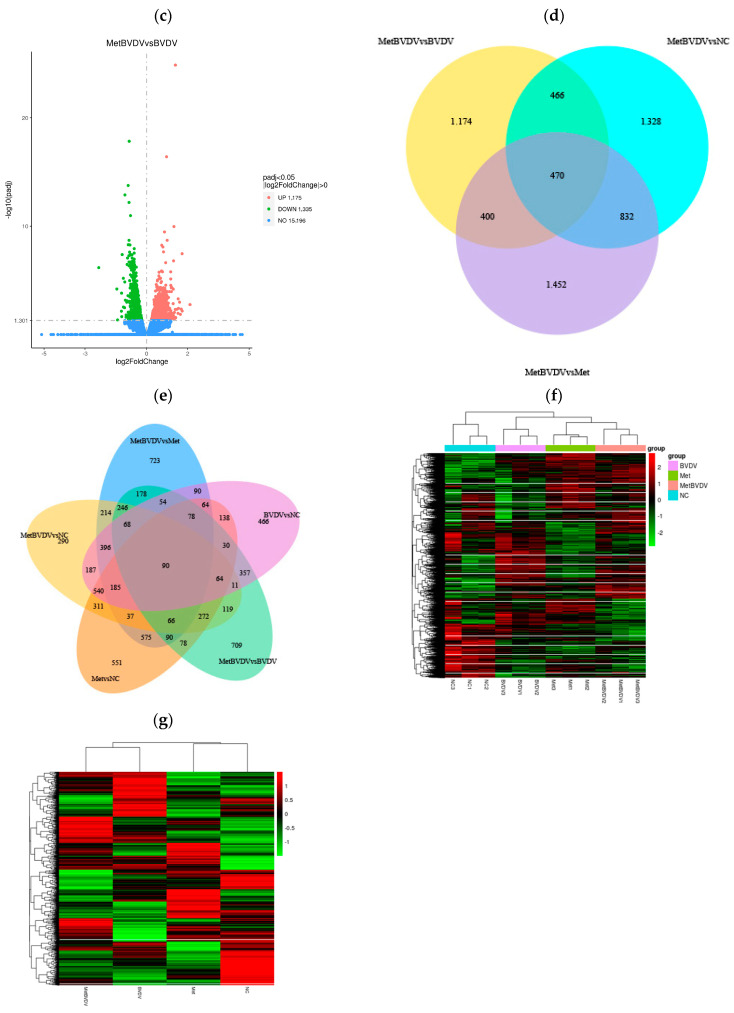
Differential gene expression analysis. (**a**): Difference comparisons depicted as histograms of combined differences in gene number statistics. Blue and gray indicate upregulated and downregulated DEGs, respectively. (**b**): DEG volcano map for the Met vs. NC groups. (**c**): Volcano map of DEGs between the MetBVDV and BVDV groups. The abscissa in the figure is the log2-fold change value, the ordinate is the −log10 padj or −log10 *p* value, and the blue dotted line represents the threshold line of the DEG screening criteria. (**d**): Venn diagram of DEGs between the MetBVDV and BVDV groups. (**e**): Venn diagram of DEGs for all combinations. (**f**): DEG clustering heatmap; the abscissa is the sample name, and the ordinate is the normalized value of the DEG FPKM. (**g**): Clustering heatmap of DEGs. Red indicates high expression, and green indicates low expression.

For the determination of the effects of Met on gene expression in BVDV-infected cells, the gene expression level was calculated via feature count v1.5.0 p3 for the gene sequence in each sample. FPKM conversion was subsequently performed to calculate the normalized gene expression level of each gene, whereas the quantitative analysis of each gene expression level was performed via RSEM v1.2.24. Differential expression analysis of the reference genes was performed via DESeq2 1.44.0, and DEGs with |log2 (FC)| ≥ 2 and corrected *p* < 0.05 were identified (Figure 3a). In the NC vs. Met comparison group, 3169 DEGs were identified; 1625 were upregulated, and 1544 were downregulated. In the MetBVDV vs. BVDV comparison group, 2510 genes were identified; 1175 were upregulated, and 1335 were downregulated.

A volcano plot was generated to visualize the distribution of DEGs for each comparison combination (Figure 3b,c). The abscissa in the graph represents the fold change in gene expression (log2 FoldChange) in the treatment and control groups, whereas the ordinate represents the genes in the treatment and control groups. The significance of the differences in expression levels is given by the −log10 padj or −log10 *p* value. In Figure 3, upregulated genes are represented by red dots, and downregulated genes are represented by green dots.

Venn diagrams can show the overlap of DEGs between different comparisons and allow for the screening of the common or unique DEGs of a certain comparison combination. We further studied the differences in gene expression induced by Met in BVDV-infected or noninfected MDBK cells by analyzing DEGs in cells with and without Met treatment and with and without BVDV infection at the same stage; we found 551 and 709 DEGs, respectively, in these groups, and 90 genes were common to all groups (Figure 3d,e).

The DEGs with similar expression patterns among the samples were clustered. As shown in Figure 3f,g, our hierarchical cluster analysis of the Met and MetBVDV groups revealed that the expression of the DEGs increased in the Met and MetBVDV groups compared with that in the NC group. Taken together, these findings indicate that BVDV infection results in dynamic increases in expression levels.

### 3.4. Enrichment Analysis of GO Terms and KEGG Pathways

**Figure 4:**
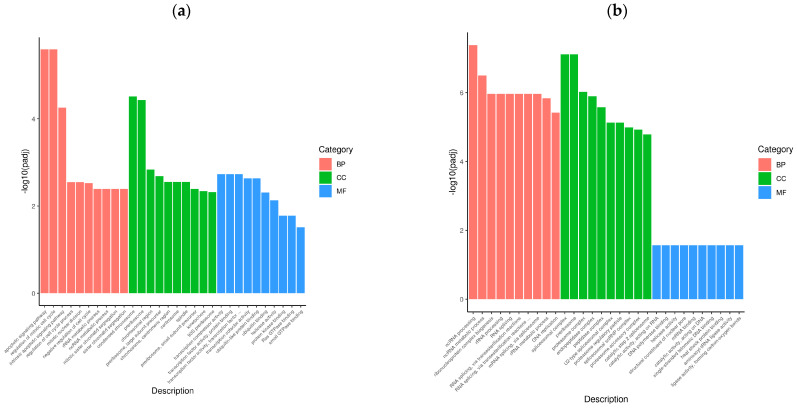

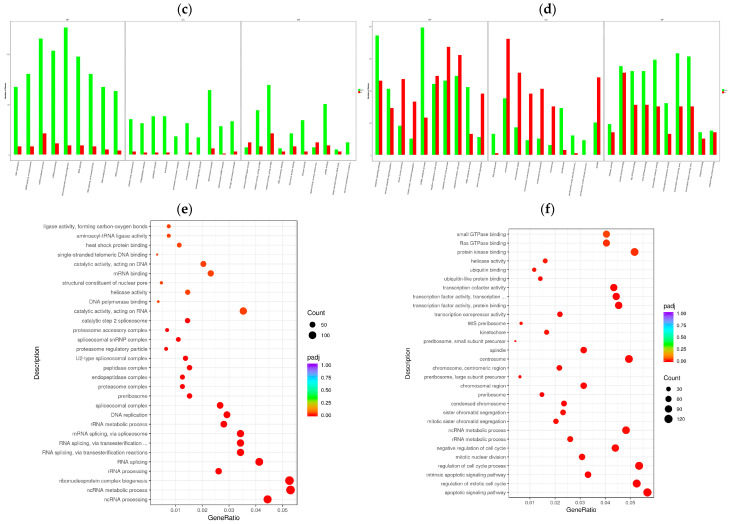
GO term enrichment analysis. (**a**): GO enrichment analysis histogram of the Met vs. NC group. (**b**): GO enrichment analysis histogram of the MetBVDV vs. BVDV group. Different colors indicate different functional classifications. (**c**): The Met vs. NC histogram drawn according to the three major categories, BPs, CCs, and MFs, and the upregulated and downregulated DEGs. (**d**): A histogram of the MetBVDV vs. BVDV groups was drawn on the basis of three major categories, as well as the upregulated and downregulated DEGs. The abscissa in the figure is the GO term, and the ordinate is the number of genes enriched in the GO term. (**e**): Scatter plot of the GO enrichment analysis results for the Met and NC groups. (**f**): Scatter plot of the GO enrichment analysis results for the MetBVDV and BVDV groups.

To determine the cellular response to BVDV infection, we performed enrichment analyses to annotate gene function and provide the most detailed information on the KEGG pathways. GOtools was used to conduct GO enrichment analysis of the DEGs among the comparison groups, and histograms were constructed with the GO terms as horizontal coordinates and the enrichment rates as vertical coordinates. The results revealed that the DEGs selected in each comparison group were associated with three categories: biological process (BP), cellular component (CC), and molecular function (MF). For GO functional enrichment, a padj less than 0.05 was used as the threshold. The DEGs selected in the Met vs. NC comparison group were significantly enriched in the ncRNA metabolic process, ribonucleoprotein complex biogenesis, rRNA processing, RNA splicing, RNA splicing via transesterification reactions, the spliceosomal complex, the proteasome complex, and catalytic activity acting on RNA. The DEGs identified in the MetBVDV vs. BVDV comparison group were significantly enriched in the apoptotic signaling pathway, the regulation of the mitotic cell cycle, the intrinsic apoptotic signaling pathway, and the negative regulation of the cell cycle, among others (Figure 4a,b). We selected the 30 most significant terms from the GO enrichment analysis results and generated a histogram according to these three major categories and the upregulated and downregulated DEGs (Figure 4c,d). We also chose 30 significant terms from the GO analysis to create scatter plots. The size of the dot represents the number of genes, and the color from red to purple represents significant enrichment (Figure 4e,f).

**Figure 5:**
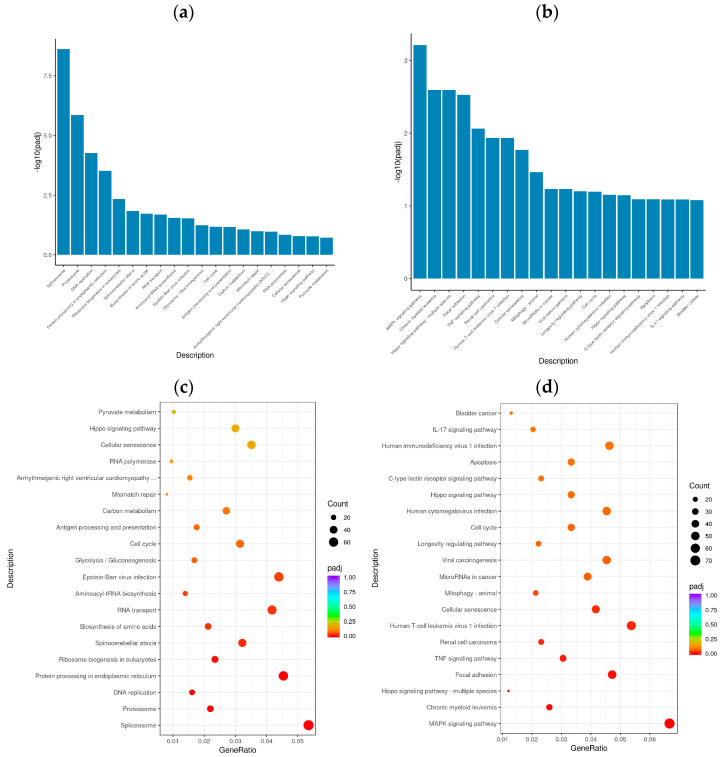
KEGG analysis. (**a**): KEGG enrichment histogram for Met vs. NC; (**b**): KEGG enrichment histogram for MetBVDV vs. BVDV. The abscissa in the figure is the KEGG; (**c**): KEGG enrichment scatter plot for Met vs. NC group. (**d**): KEGG enrichment scatter plot for MetBVDV vs. BVDV; in the figure, the abscissa represents the ratio of the number of DEGs annotated to the KEGG pathway to the total number of DEGs, and the ordinate represents the KEGG pathway.

In organisms, genes do not function alone; rather, they often interact with each other to form various metabolic and signal transduction pathways. KEGG pathway enrichment analysis helped elucidate the biological functions of the DEGs and the biological systems of active cells after BVDV infection. KOBAS 2.0 was used to perform a KEGG pathway analysis of the DEGs of the different comparison groups. The KEGG pathway was used as the abscissa, and the enrichment rate was used as the ordinate to construct a histogram. KEGG pathway enrichment analysis revealed 20 significantly enriched signaling pathways in the Met vs. NC and MetBVDV vs. BVDV comparison groups (Figure 5a,b). Significant enrichment for the Met vs. NC comparison group was observed in the spliceosome signal transduction pathway, the protein processing signaling pathway in the endoplasmic reticulum, the aminoacyl-tRNA biosynthesis signaling pathway, the cell cycle signaling pathway, cysteine and methionine metabolism, NF-κB, sphingolipid, MAPK, the autophagy signaling pathway, and endocytosis, whereas for the MetBVDV vs. BVDV comparison group, significant enrichment was indicated for the Hippo, TNF, mitochondrial autophagy, the C-type lectin receptor, and the PI3K-Akt signaling pathways (Figure 5c,d). Thus, the GO and KEGG pathway enrichment analyses identified at least 20 signaling pathways.

### 3.5. GSEA

**Figure 6:**
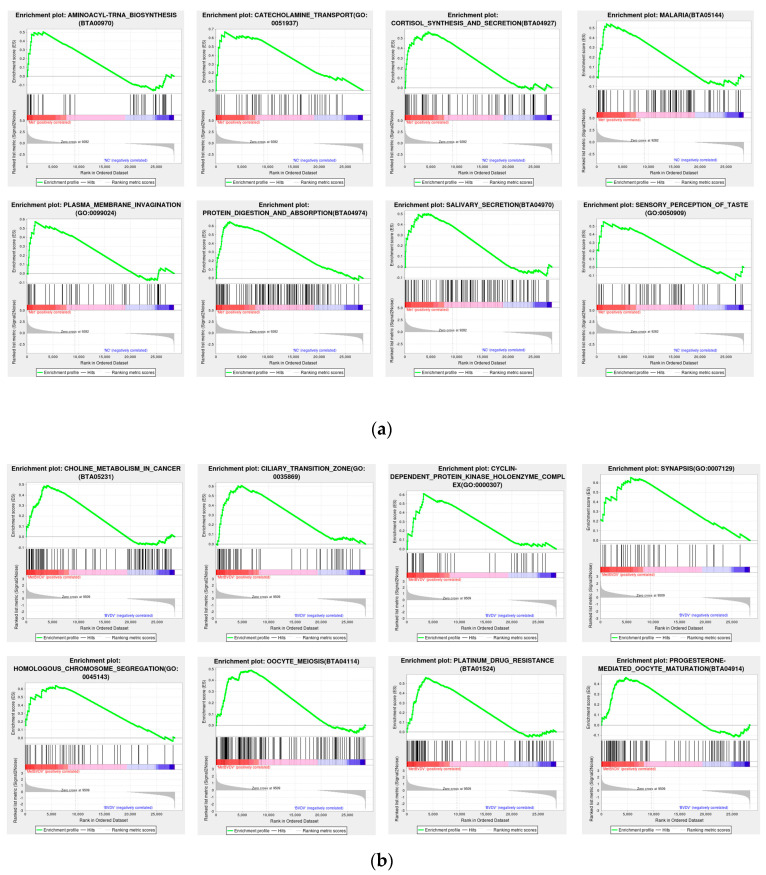
GSEA. (**a**): GSEA chart of the Met vs. NC groups. (**b**): GSEA chart of the Met vs. NC groups. The figure is divided into three parts. The first part is the line chart of the gene enrichment score. The highest peak is the enrichment score (ES). Gene sets with a particularly obvious peak on the far left or right are usually the gene sets of interest. The middle part is a hit, and each line represents a gene in the gene set and its rank in the gene list. The bottom part is the rank value distribution map of all genes, showing the matrix of genes and phenotypes, where red indicates a positive correlation with the first phenotype (ISO) and blue indicates high expression in ISO. The second table type (control) shows positive correlations, with high expression observed in the control. The distribution of gene expression in all the samples in the gene set is also displayed in the form of heatmaps. Each row represents a gene, where the gene expression level changes from low to high and the color transitions from blue to red.

To prevent the omission of genes that were not significantly differentially expressed yet had significant biological importance, we employed GSEA. GSEA utilizes a predefined gene set to rank the genes on the basis of their differential expression levels in two types of samples and subsequently examines whether the preset gene set is positioned at the top of the ranking list or enriched at the bottom. The results for the Met vs. NC group revealed that the addition of Met was related to aminoacyl-tRNA biosynthesis (BTA00970), catecholamine transport (GO: 0051937), cortisol synthesis and secretion (BTA04927), malaria (BTA05144), plasma membrane invagination (GO: 0099024), protein digestion and absorption (BTA04974), salivary secretion (BTA04970), and sensory perception of taste (GO: 005090). The results of the MetBVDV vs. BVDV comparison revealed that combined Met treatment and BVDV infection, compared with BVDV infection alone, was related to choline metabolism in cancer (BTA05231), the ciliary transition zone (GO: 0035869), the cyclin-dependent protein kinase holoenzyme complex (GO: 0000307), synapsis (GO: 0007129), homologous chromosome segregation (GO: 0045143), oocyte meiosis (BTA04114), platinum drug resistance (BTA01524), and progesterone-mediated oocyte maturation (BTA04914) (Figure 6a,b). All of the above results indicated that Met significantly altered several vital pathways.

### 3.6. PPI Network Analysis of DEGs

**Figure 7:**
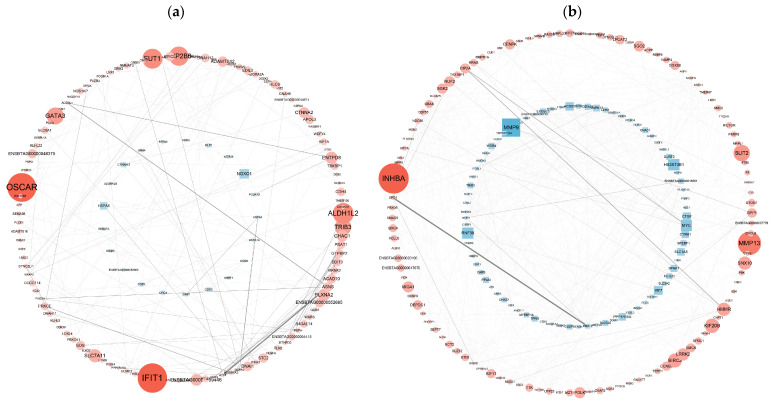
PPI network analysis of the DEGs. (**a**): The differential gene expression thresholds for the Met vs. NC comparison were a multiplier greater than two and *p* < 0.01. (**b**): The differential gene expression thresholds for the MetBVDV vs. BVDV comparison were multipliers greater than 1.5 and *p* < 0.01. A circle indicates an upregulated gene, and a rectangle indicates a downregulated gene. The color of the node represents the fold change (a darker red indicates greater upregulation, and a darker blue indicates greater downregulation), and the thickness of the line represents edge-between-ness, where a thicker line indicates that the interaction is more important in the network.

To determine the biological relevance of the DEGs, we performed PPI network analyses of the DEGs with the Cytoscape 3.8.2 database. The PPI network was visualized via the NetworkX package in Python. PPI analyses of DEGs in the Met vs. NC and MetBVDV vs. BVDV groups were based on DEGs with FDR ≤ 0.05 and |log2 FC| ≥ 2. PPI network analysis revealed interactions among 134 genes among the DEGs from the control Met vs. NC comparison and revealed that the DEGs ALDH1L2, SLC34A2, INHBE, ESRP1, and TRIB3 were important in maintaining the close connections of the entire network. The analysis also revealed interactions among 188 DEGs in the MetBVDV vs. BVDV comparison group and revealed that the DEGs HSD3B1, SPP1, PSMB10, RAD18, NUP107, DPM2, SLC38A7, and NFXL1 played important roles in maintaining the close connections of the entire network. The DEGs GCC2, SUGT1, HDHD5, MRPL17, MAP2, DLG3, SSRP1, SERTAD1, SPN, and TNFAIP8 play key roles in signal transduction in a variety of cell activities (including the cell cycle, apoptosis, gene expression regulation, and disease development). These results indicate that BVDV promotes its own replication in host cells by interfering with the expression of these genes. Met may downregulate the expression of autophagy-related genes through the autophagy pathway, thereby inhibiting BVDV replication (Figure 7a,b).

### 3.7. qPCR Analysis

**Figure 8:**
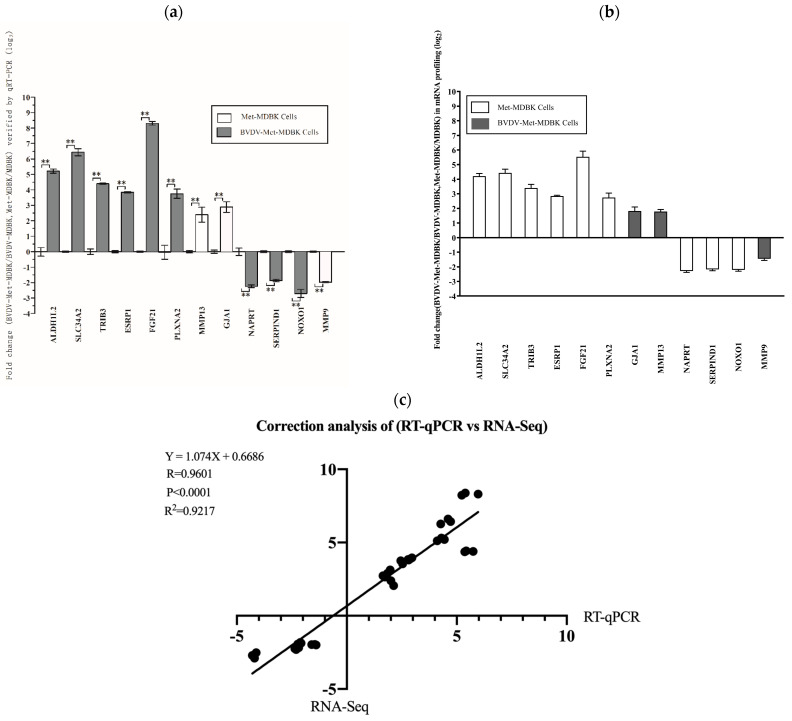
Relative expression of DEGs validated by qPCR. (**a**): Expression levels of 12 genes validated by qPCR. The GAPDH gene was used as an internal control, and the relative quantity of gene expression (fold change) of each gene was calculated via the comparative 2^−ΔΔCT^ method. (**b**): Fold change in the expression of 12 randomly selected DEGs according to RNA-seq-based transcriptomic profiling of MDBK cells. The data are expressed as the log2 (fold change) and are shown as the mean ± SD. *n* = 3. (**c**): Correlations of the fold changes according to the data obtained via qPCR (*x*-axis) with the RNA-seq platform (*y*-axis). The asterisks indicate statistical significance according to the Student’s *t* test (**, *p* < 0.01).

To validate the reproducibility and repeatability of the DEGs identified from transcriptome sequencing, we randomly selected twelve genes for qPCR analysis: SLC34A2, FGF21, ALDH1L2, TRIB3, PLXNA2, ESRP1, GJA1, MMP13, NAPRT, SERPIND1, NOXO1, and MMP9. Figure 8a shows that these genes were significantly differentially expressed, as determined by RNA-seq, and the DEGs obtained from transcriptome sequencing were deemed reliable (Figure 8b). Correlations between the RNA-seq and qPCR data were determined via scatter plots of the log2-fold change values. As shown in Figure 8c, the qPCR results were highly correlated with the RNA-seq results (R^2^ = 0.9217). These results showed that the transcriptomic sequencing results were reproducible and repeatable.

## 4. Discussion

Diseases caused by BVDV remain among the most important health problems. To date, no specific therapeutic agent with activity against BVDV is available, and PI has been observed in an increasing number of cattle. Therefore, identification of new strategies to control BVDV infections is essential. Our findings indicate that Met has good potential as an antiviral drug. We found that Met inhibited BVDV and had antiviral effects at 10 mM. The BVDV life cycle is composed of four stages: attachment, internalization, replication, and release. Met significantly suppressed every stage of BVDV replication.

In the present study, an analysis of the transcriptomic differences before and after BVDV infection in MDBK cells that were untreated or pretreatment with Met revealed that Met activated the MAPK signaling pathway and altered the host metabolic network. PPI network analysis revealed interactions among 134 genes among the DEGs from the control Met vs. NC comparison and revealed that the DEGs SLC34A2 and TRIB3 played important roles in maintaining the close connections of the entire network. SLC34A2 interacts with syntaxin 17 (STX17) to activate autophagy [18,19]. Blocking autophagic flux subsequently leads to defects in the clearance of ubiquitinated proteins [20]. These DEGs indicated that Met significantly reduced BVDV infection via autophagy.

In the intracellular replication stage after infection, BVDV infection downregulated the expression of several host antiviral genes, whereas the BVDV infection of Met-treated MDBK cells downregulated the expression of genes involved in mitochondrial autophagy, indicating that this pathway is important for the inhibition of BVDV by Met. The MetBVDV vs. BVDV comparison revealed 188 DEGs, including HSD3B1 and SLC38A7. HSD3B1 transcription increases autophagic ATG expression [21], and SLC38A7 is a lysosomal membrane protein that activates macroautophagy [22]. DEGs were also enriched in several key biological processes or pathways, such as the cell adhesion molecule (CAM), MAPK, and autophagy pathways [23,24]. The MAPK extracellular signal regulated kinase (ERK) pathway is a node for signal convergence and is an important transmitter of signals from the cell surface to the inside of the nucleus [25], such as growth and proliferation, cell differentiation, cell movement, and death [26,27]. After Met treatment, 52 genes enriched in the MAPK pathway were identified, 28 of which were upregulated and 24 of which were downregulated. Compared with those in infected cells not treated with Met, 11 genes in the MAPK signaling pathway in BVDV-infected MDBK cells were upregulated, and the expression of 12 genes was downregulated. Therefore, Met may affect BVDV replication through the MAPK signaling pathway.

In addition, HCV has the same structural organization, genomic characteristics and common steps of viral replication as BVDV and can attach to the same host-cell low-density lipoprotein (LDL) receptor as BVDV. However, their genome sizes [28] are different. Therefore, BVDV is widely used as an alternative to HCV [29,30]. Mitophagy is the selective degradation of mitochondria through autophagy [31]. As a stress-related process in eukaryotic cells, mitophagy involves the degradation and circulation of many signaling pathways and biological macromolecules in the cell and in damaged organelles. Viral infections, such as those caused by HCV, can cause mitochondrial autophagy in many ways. For example, Kim et al. [29] reported that the HCV infection of hepatocytes induces PINK1-PRKN-dependent mitochondrial autophagy and that the activation of mitochondrial autophagy can increase viral replication. Jassey et al. [32] reported that the nonstructural protein 5A of HCV activates mitochondrial autophagy. In addition, HCV can mediate mitochondrial autophagy by activating dynamic related protein 1 (Drp1), reducing host cell apoptosis, and facilitating chronic HCV infection. Met effectively inhibited flavivirus replication in vivo and in vitro in a previous study [33], but the mechanism of action is still unclear. These studies indicate that mitochondrial autophagy is closely related to the pathogenic mechanism of HCV. Further research by Kim et al. [29] revealed that the ginsenoside Rg3 can be used as a candidate anti-HCV drug that inhibits the spread of HCV by regulating the abnormal mitochondrial division and mitochondrial autophagy caused by HCV.

In future work, we will study the mechanism by which Met acts on ATG10 to affect cell autophagy and, in turn, affects the replication of BVDV. We will also explore the effects of Met on other viruses. BVDV is a globally distributed animal pathogen that causes economic losses in the cattle industry but also increases the damage caused by other pathogens in secondary infections. The mechanism of BVDV infection and pathogenicity is complex but requires successful escape from the host immune system through molecular mechanisms initiated by BVDV. The present study, through transcriptomic sequencing and analysis, provides clues regarding the effect of Met on gene expression in MDBK cells before and after BVDV infection and can guide studies on the interaction between BVDV and the host and the inhibition of BVDV replication by Met.

In this study, we characterized the transcriptomic profile of Met-treated or untreated MDBK cells with or without BVDV infection. The dynamic changes in DEGs caused by Met treatment helped elucidate the molecular mechanisms of BVDV, Met and host interactions.

## 5. Conclusions

Our results revealed that BVDV infection induced alterations in the host metabolic network, while Met treatment activated the AMPK pathway in cells, and the autophagy pathway in the Met-treated MDBK cells was activated after infection with BVDV. These results provide unique insights for future research on the mechanism of interaction between BVDV and Met and can be used as the basis for strategies for the prevention and treatment of BVD. Clarifying the mechanism and target of Met is therefore an important direction in the search for anti-BVDV drugs.

## Figures and Tables

**Table 1:** Primers for qPCR analysis.

Gene ID	Gene Name	GenBank	Primers (5′→3′)
gene1545	SLC34A2	NM_174661.2	CTGCGTCTTCCAAGGGATTG
			CCCAGCTACCTTTCCTCCAA
gene11624	FGF21	XM_024979245.1	TTCCTTGAGGAGGACGCTTT
			CCTTGCACATGGACTCACAG
gene6326	ALDH1L2	NM_001191391.1	TTTCTGCCACAGAACAAGGC
			ATGGGTGGGATGAGGGAAAG
gene17007	TRIB3	NM_001076103.1	GGAGAACCTGGAAGATGCCT
			GGCCAGCATGGTAAAGAGTG
gene1173	PLXNA2	NM_001206657.2	GGAACCTGACGGAGGTAGAG
			CTAGCCCAAACCAGTCCTGA
gene21838	ESRP1	NM_001193002.1	ACGGAGGACTGCAAAGAAGA
			TTAAACTGTCGGAGCGCTTG
gene1835	GJA1	NM_174068.2	GCCTTCTTGCTGATCCAGTG
			GCCGAGAAAGGAAACAGTCC
gene15059	MMP13	NM_174389.2	AACGCCAGACAAATGTGACC
			CTTCAACCTGCTGAGGATGC
gene14642	NAPRT	XM_027560533.1	AGGTGAACGTCATTGGCATC
			CCAGGCAATGTCTGCTTCTC
gene13973	SERPIND1	NM_001105046.2	GCCCTTCCTGTTCCTCATCT
			AGCTCTTGGTGGTTGTCAGA
gene20369	NOXO1	XM_027527024.1	GGCCCTTCTCCAACATCTCT
			GGCCTGGTAGGGTACAAAGT
gene20676	MMP9	NM_174744.2	CACGCACGACATCTTTCAGT
			TCACGTAGCCCACATAGTCC
	GAPDH	XM_014482068.1	AAGGTCGGAGTGAACGGATT
			CGTTCTCTGCCTTGACTGTG

**Table 2:** Transcriptomic sequencing data.

Sample	Library	Raw_Reads	Clean_Reads	Clean_Bases	Error_Rate	Q20	Q30	GC_pct
BVDV1	FRAS210001071-1r	42743418	41200374	6.18 G	0.02	98.09	94.76	54.58
BVDV2	FRAS210001072-1r	42083470	40540156	6.08 G	0.03	97.57	93.62	54.9
BVDV3	FRAS210001073-1r	44209666	42370430	6.36 G	0.02	97.98	94.58	55.77
Met1	FRAS210001074-1r	54087394	52340500	7.85 G	0.02	97.91	94.31	53.54
Met2	FRAS210001075-1r	47426060	45846420	6.88 G	0.03	97.88	94.31	53.91
Met3	FRAS210001076-1r	43909684	41876892	6.28 G	0.02	97.95	94.45	53.66
MetBVDV1	FRAS210001077-1r	42367072	40441680	6.07 G	0.02	98.02	94.63	53.28
MetBVDV2	FRAS210001078-1r	40551430	39255780	5.89 G	0.02	98.01	94.56	53.88
MetBVDV3	FRAS210001079-1r	41821636	40469088	6.07 G	0.02	98.11	94.74	53.05
NC1	FRAS210001080-1r	41858954	39774782	5.97 G	0.02	98.02	94.55	53.09
NC2	FRAS210001081-1r	43160394	41215010	6.18 G	0.02	98.01	94.56	53.15
NC3	FRAS210001082-1r	45915446	44639786	6.7 G	0.02	97.94	94.42	54.53

## Data Availability

No new data were created or analyzed in this study. Data sharing is not applicable to this article.
